# Cannabis use patterns among emerging adults in California who use tobacco: Differences by sexual identity, gender identity, and sex assigned at birth

**DOI:** 10.1016/j.abrep.2025.100624

**Published:** 2025-07-14

**Authors:** Evan A. Krueger, Luisita Cordero, Chenglin Hong, Risa Flynn, Ian W. Holloway

**Affiliations:** a School of Social Work, Tulane University, New Orleans, LA 70112, USA; b Department of Social Welfare, Luskin School of Public Affairs, University of California, Los Angeles, CA 90095, USA; c School of Social Work, University of Connecticut, Hartford, CT 06103, USA; d Los Angeles LGBT Center, Los Angeles, CA 90028, USA

**Keywords:** Cannabis, Co-use, Emerging adults, Gender minority, Sexual minority

## Abstract

**Background:**

Sexual and/or gender minority (SGM) emerging adults use both cannabis and tobacco products at higher rates than their non-SGM peers. Co-use of cannabis and tobacco is of increasing public health concern, yet relatively little is known about co-use among SGM emerging adults.

**Methods:**

Data were from a cross-sectional survey (2020 – 2021) of 1,467 SGM (n = 868) and non-SGM (n = 623) emerging adults from California. To be eligible, participants reported using at least one tobacco product in the prior 30 days. We assessed differences in lifetime and recent (past 3-month) cannabis use, and in concurrent use of both substances, across six groups: cisgender heterosexual, cisgender sexual minority (SM), transgender/gender non-conforming (TGNC) participants assigned female at birth (AFAB), and cisgender heterosexual, cisgender SM, and TGNC participants assigned male at birth (AMAB).

**Results:**

Among AFAB, cisgender SM and TGNC participants (vs. cisgender heterosexual) had higher odds of lifetime (cisgender SM: aOR = 2.38, 95 % CI = 1.54, 3.72; TGNC: aOR = 1.97, 95 % CI = 1.17, 3.40) and recent cannabis use (cisgender SM: aOR = 1.72, 95 % CI = 1.29, 2.30; TGNC: aOR = 1.83, 95 % CI = 1.29, 2.61), and of concurrent use (cisgender SM: aOR = 1.49, 95 % CI = 1.11, 2.00; TGNC: aOR = 1.98, 95 % CI = 1.39, 2.82). No differences in cannabis and tobacco co-use were noted among AMAB.

**Conclusion:**

Cisgender SM and TGNC AFAB are at high risk for cannabis and tobacco co-use. Prevention and harm reduction interventions should be tailored to these groups.

## Highlights

• SM (vs. heterosexual) AFAB participants had higher odds of cannabis use.
• TGNC (vs. cisgender) AFAB participants had higher odds of cannabis use.
• SM (vs. heterosexual) AFAB participants had higher odds of concurrent use.
• TGNC (vs. cisgender) AFAB participants had higher odds of concurrent use.
• No differences in cannabis use and co-use were noted among AMAB participants.

## 1 Introduction

Cannabis is one of the most widely used substances among emerging adults in the United States (Centers for Disease Control and Prevention., 2021, Hasin and Walsh, 2021, National Institute on Drug Abuse, 2020, Wadsworth and Hammond, 2019), with the prevalence of use having increased in recent years. For instance, among emerging adults in the Monitoring the Future study (MTF; participants were aged 19 – 30), 29 % reported past-month cannabis use in 2021, compared to 17 % in 2011 (National Institutes of Health, 2022). Further, despite declining cigarette use nationally, emerging adults’ use of e-cigarettes and other non-cigarette tobacco products (e.g., nicotine pouches, cigars and cigarillos, hookah), has also increased; in MTF, 16 % of emerging adults reported e-cigarette use in 2021, compared to 6 % in 2017 (National Institutes of Health, 2022). Cannabis and tobacco co-use refers to the concurrent use of both products and/or use of both products within a given timeframe (National Cancer Institute, 2023), and is an area for public health concern because those who use both products report greater dependence on each product (Akbar et al., 2019, Fairman, 2015, Strong et al., 2018) and also have a harder time successfully quitting use of either product (Driezen et al., 2022, Weinberger et al., 2020). Cannabis and tobacco co-use is increasingly common among emerging adults (Cohn et al., 2019, Wheldon et al., 2023); in the Population Assessment of Tobacco and Health Study (PATH; participants were aged 18 – 24), 47.9 % of emerging adults who reported past 30-day tobacco use also reported using cannabis in the prior 30 days (Cohn & Chen, 2022).

Sexual and/or gender minority (SGM; e.g., lesbian, gay, bisexual, transgender-identified) emerging adults use both cannabis and tobacco products at higher rates than non-SGM emerging adults (Dai, 2017, Delahanty et al., 2019, Dunbar et al., 2022, Harlow et al., 2023, Krueger et al., 2020, Romm et al., 2024, Romm et al., 2023, Sawyer et al., 2022). However, there are few studies on co-use among SGM, and they are limited to sexual minority (SM; e.g., lesbian, gay, bisexual) populations. In the PATH Study, Wave 5, 27.6 % of SM women and 21.5 % of SM men reported use of both products in the past 30 days (Romm et al., 2024). In an online comparative study of women, SM women had higher odds (aOR = 1.74) of cannabis and tobacco co-use, compared to heterosexual women (Ehlke et al., 2023). In a daily diary study, SM emerging adults had greater odds of same-day co-use (aOR = 2.05), compared to heterosexuals (Nguyen et al., 2021). To our knowledge, there are no studies on gender minority (e.g., transgender, non-binary) cannabis and tobacco co-use.

The bulk of SGM cannabis and tobacco use research compares rates of use between SGM and non-SGM participants broadly, without attention to potentially important subgroup differences in use. SGM substance use patterns commonly vary by sex assigned at birth (i.e., between those assigned female vs. male at birth), with, for instance, disparities evident between SM and heterosexual women across a number of substances, and fewer disparities noted among men (Austin et al., 2004, Caputi, 2018, Delahanty et al., 2019). However, little is known about sex differences in SM cannabis use, or in co-use, and there are no known studies examining sex differences in co-use among gender minority emerging adults (i.e., between transgender/gender non-conforming [TGNC] persons assigned female at birth [AFAB] and those assigned male at birth [AMAB]). While sampling limitations commonly preclude researchers from examining sex differences in SGM substance use, understanding those differences as they relate to co-use is important for developing effective and tailored prevention and harm reduction interventions.

### 1.1 The current study

We assessed the prevalence of cannabis use among a large, diverse sample of SGM and non-SGM emerging adults from California who use tobacco. We examined differences in use across six analytic subgroups: cisgender heterosexual AFAB, cisgender SM AFAB, and TGNC AFAB, and cisgender heterosexual AMAB, cisgender SM AMAB, and TGNC AMAB.

## 2 Methods

### 2.1 Sample

Data were from a cross-sectional survey study of 1,467 emerging adults (ages 18 – 29) from California designed to compare tobacco and cannabis use behaviors between SGM (n = 857) and non-SGM (n = 610) participants. To be eligible for the study, participants had to report using a nicotine or tobacco product in the prior 30 days, be between the ages of 18–29, and reside in California. Details of the recruitment strategy have been published elsewhere (Krueger et al., 2023). Briefly, participants were recruited between March 2020 and August 2021 from a variety of locations, including in-person from the Los Angeles LGBT Center, and through online ads placed on social media websites (e.g. Instagram) and dating websites (e.g. Grindr), via direct mailer, and from an existing online panel on Qualtrics; 41.6 % (n = 611) of the sample was recruited from Qualtrics and 58.4 % (n = 856) was recruited from across the other sources (Krueger et al., 2023). The study was approved by the University of California, Los Angeles Office of the Human Research Protection Program.

### 2.2 Variables

#### 2.2.1 Cannabis and tobacco co-use

Lifetime and recent cannabis and tobacco use, and concurrent use (use of cannabis and tobacco at the same time) were assessed. Participants were first asked “In your life, which of the following substances have you ever used? (non-medicinal use only).” Response options included “cannabis (marijuana, pot, grass, hash, etc.).” Those reporting lifetime use were asked a follow-up question to assess recent use: (“In the past 3 months, how often have you used the substances you mentioned?”: never, once or twice, monthly, weekly, daily or almost daily). Concurrent use was assessed with the question, “How often have you used tobacco when you have also been using marijuana/cannabis?”: usually, sometimes, never. Those selecting “usually” or “sometimes” were coded as concurrently using cannabis and tobacco products. Participants reporting concurrent use were asked which tobacco products they had used: cigarettes, e-cigarettes, another tobacco product (cigars, cigarillos, little filtered cigars, or cheroots; regular pipes full of tobacco; hookah; smokeless tobacco; dissolvable tobacco), or multiple product types. Tobacco product categories were not mutually exclusive (e.g., a participant reporting both cigarette and e-cigarette use would be coded as using cigarettes, e-cigarettes, and multiple product types). Finally, use of blunts (cannabis rolled in a tobacco leaf) in the past 30 days was also assessed using the question, “During the past 30 days, did you smoke part or all of a cigar with marijuana/cannabis in it, which is sometimes called a blunt?” (yes, no).

#### 2.2.2 Sexual identity, gender identity, and sex assigned at birth

Participants reported their sexual orientation (“which of the following best describes your current sexual orientation?”: straight or heterosexual, lesbian, gay, bisexual, queer, pansexual, same-gender loving, asexual, sexual orientation not listed here), and were categorized as heterosexual or SM (any identity other than “straight or heterosexual”). Participants also reported their current gender identity (“If you had to choose only one of the following terms, which best describes your current gender identity?”: cisgender male/man, cisgender female/woman, transgender male/man, transgender female/woman, genderqueer/ gender non-binary/ gender non-conforming/ gender fluid, gender identity not listed here). Participants were categorized as either cisgender (cisgender male/man, cisgender female/woman) or gender minorities (any identity other than cisgender male/man or cisgender female/woman). Finally, participants reported their sex assigned at birth (“What sex were you assigned at birth, on your original birth certificate?”) as either male or female. Based on participants’ responses to these three items, participants were assigned to one of six mutually exclusive categories: cisgender heterosexual, cisgender SM, and TGNC participants assigned female at birth (AFAB), and cisgender heterosexual, cisgender SM, and TGNC participants assigned male at birth (AMAB).

#### 2.2.3 Covariates

Sociodemographic covariates included race/ethnicity (White, Asian, Black/African American, Hispanic/Latino or Spanish origin, multiracial, other), country of birth (the United States, outside the United States), educational attainment (less than a GED/high school diploma, GED/high school diploma, some college/Associate’s degree, Bachelor’s degree or higher), employment (full-time, part-time, self-employed, other, student, and unemployed; those indicating more than one employment status were assigned the highest-ranking option according to the order listed here), housing stability (unstable, stable; coded as unstable if any of the following were reported alone or in combination: on the street, in a car, in an abandoned building, in a park, or a place that is not a house, apartment, shelter, or other housing; substance abuse treatment center or sober living; in a shelter; in a group home facility), marital status (married/living with a partner, single, divorced/separated/widowed/other), and age. Additional covariates included past-month psychological distress [assessed using the Kessler-6 scale (Kessler, n.d.)] and social support [assessed using the multidimensional scale of perceived social support (Zimet et al., 1988)].

### 2.3 Data Analysis

Sociodemographic characteristics were first calculated for the full sample, and separately by sexual/gender identity and sex assigned at birth. Differences were tested across sexual/gender identities using chi-square (categorical) and ANOVA (continuous) tests. Next, prevalence estimates of lifetime cannabis use, recent cannabis use, and concurrent use were obtained, and sexual/gender identity and sex assigned at birth differences were tested using chi-square tests. Differences in past 30-day blunt use were also assessed across groups using chi-square tests. However, given the different time frames (i.e., past 30-days for blunts vs. past 3 months for recent cannabis use), results for blunt use are reported separately. Then, associations between sexual/gender identities and the cannabis use outcomes were estimated using multiple logistic (lifetime use) and generalized ordered logit (recent use, concurrent use) regressions, controlling for covariates; to account for multiple comparisons, Benjamini-Hochberg corrected *p*-values were used. Finally, among those reporting concurrent use, the specific tobacco products used were compared across sexual/gender identity groups using chi-square tests. Listwise deletion was employed, as the extent of missingness was minimal and did not meaningfully impact the sample size or key variables. Generalized ordinal logit models were performed using Stata and all other analyses were conducted using R.

## 3 Results

### 3.1 Sociodemographic characteristics of the sample

Shown in Table 1, several sociodemographic characteristics varied across sexual/gender identity subgroups. Among AFAB participants, race/ethnicity varied across identity groups, with, for instance, 39.8 % of cisgender SM participants identifying as White, compared to 30.0 % of TGNC participants (p < 0.001). Housing stability also varied across groups, with 9.5 % of TGNC participants reporting unstable housing, compared to 4.5 % of cisgender SM participants (p = 0.042).Table 2..

**Table 1:** Sociodemographic Characteristics of the Sample.

		**Assigned Female at Birth**				**Assigned Male at Birth**			
	**Full Sample**	**Cisgender heterosexual**	**Cisgender SM**	**TGNC**	**P-Value**	**Cisgender heterosexual**	**Cisgender SM**	**TGNC**	**P-Value**
	N = 1467	N = 302	N = 379	N = 190		N = 308	N = 204	N = 84	
**Race/Ethnicity, n (%)**					<0.001				0.004
White	528 (36.0)	103 (34.1)	151 (39.8)	57 (30.0)		103 (33.4)	85 (41.7)	29 (34.5)	
Asian	230 (15.7)	57 (18.9)	45 (11.9)	21 (11.1)		72 (23.4)	29 (14.2)	6 (7.1)	
Black	83 (5.7)	9 (3.0)	19 (5.0)	14 (7.4)		(21) 6.8	12 (5.9)	8 (9.5)	
Hispanic/Latinx	323 (22.0)	76 (25.2)	86 (22.7)	35 (18.4)		65 (21.1)	45 (22.1)	16 (19.0)	
Multiracial	254 (17.3)	43 (14.2)	68 (17.9)	61 (32.1)		34 (11.0)	27 (13.2)	21 (25.0)	
Other	49 (3.3)	14 (4.6)	10 (2.6)	2 (1.1)		13 (4.2)	6 (2.9)	4 (4.8)	
**Country of Birth, n (%)**					0.164				0.043
Outside the United States	121 (8.2)	23 (7.6)	19 (5.0)	17 (8.9)		26 (8.4)	21 (10.3)	15 (17.9)	
United States	1,346 (91.8)	279 (92.4)	360 (95.0)	173 (91.1)		282 (91.6)	183 (89.7)	69 (82.1)	
**Educational Attainment, n (%)**					0.885				0.028
Less than GED/HS diploma	53 (3.6)	11 (3.6)	15 (4.0)	11 (5.8)		9 (2.9)	4 (2.0)	3 (3.6)	
GED/HS diploma	298 (20.3)	62 (20.5)	72 (19.0)	39 (20.5)		77 (25.0)	26 (12.7)	22 (26.2)	
Some college/Associates	719 (48.4)	143 (47.4)	191 (50.4)	89 (46.8)		141 (45.8)	110 (53.9)	36 (42.9)	
Bachelor's degree or higher	406 (27.7)	86 (28.5)	101 (26.6)	51 (26.8)		81 (26.3)	64 (31.4)	23 (27.4)	
**Employment Status, n (%)**					0.186				0.113
Full time	430 (29.3)	84 (27.8)	83 (21.9)	55 (28.9)		120 (39.0)	67 (32.8)	21 (25.0)	
Part time	335 (22.8)	62 (20.5)	99 (26.1)	50 (26.3)		52 (16.9)	48 (23.5)	24 (28.6)	
Self-Employed	93 (6.3)	11 (3.6)	28 (7.4)	9 (4.7)		26 (8.4)	11 (5.4)	8 (9.5)	
Other	28 (1.9)	12 (4.0)	10 (2.6)	4 (2.1)		2 (0.6)	0 (0.0)	0 (0.0)	
Student	311 (21.2)	78 (25.8)	93 (24.5)	39 (20.5)		53 (17.2)	37 (18.1)	11 (13.1)	
Unemployed	270 (18.4)	55 (18.2)	66 (17.4)	33 (17.4)		55 (17.9)	41 (20.1)	20 (23.8)	
**Housing Stability, n (%)**					0.042				<0.001
Unstable	82 (5.6)	15 (5.0)	17 (4.5)	18 (9.5)		11 (3.6)	9 (4.4)	12 (14.3)	
Stable	1,385 (94.4)	287 (95.0)	362 (95.5)	172 (90.5)		297 (96.4)	195 (95.6)	72 (85.7)	
**Marital Status, n (%)**					0.746				0.342
Married/Living with Partner	424 (28.9)	100 (33.1)	134 (35.4)	63 (33.2)		75 (24.4)	37 (18.1)	15 (17.9)	
Single	1,013 (69.0)	195 (64.6)	240 (63.3)	125 (65.8)		227 (73.7)	160 (78.4)	66 (78.6)	
Divorced/ Separated/ Widowed/ Other	30 (2.0)	7 (2.3)	5 (1.3)	2 (1.1)		6 (1.9)	7 (3.4)	3 (3.6)	
**Age, mean**	23.0	23.0	22.9	23.0	0.988	23.6	23.7	22.9	0.195

Note. Differences were tested across sexual/gender identities using chi-square (categorical) and ANOVA (continuous) tests.

**Table 2:** Past 30-day Blunt Use by Sexual Orientation and Gender Identity.

	**Assigned Female at Birth**		**Assigned Male at Birth**	
	Past 30-Day Blunt Use (%)	P-Value	Past 30-Day Blunt Use (%)	P-Value
		0.004		0.127
Cisgender heterosexual	28.4		56.8	
Cisgender SM	44.6		30.3	
TGNC	27.1		12.8	

Note. Differences were tested across sexual/gender identities using chi-square tests.

Among AMAB participants, race/ethnicity also varied across identity groups; 41.7 % of cisgender SM participants identifying as White, compared to 33.4 % of cisgender heterosexual participants (p = 0.004). Further, 17.9 % of TGNC participants reported being born outside the United States, compared to 8.4 % of cisgender heterosexual participants (p = 0.043). In terms of educational attainment, 31.4 % of cisgender SM AMAB participants completed a bachelor’s degree or higher, compared to 26.3 % of cisgender heterosexual AMAB participants (p = 0.028). Finally, housing stability also varied; 14.3 % of TGNC AMAB participants reporting being unstably housed, compared to 3.6 % of cisgender heterosexual AMAB participants (p < 0.001).

### 3.2 Sexual/gender identity differences in cannabis use and concurrent tobacco use

**Fig. 1:**
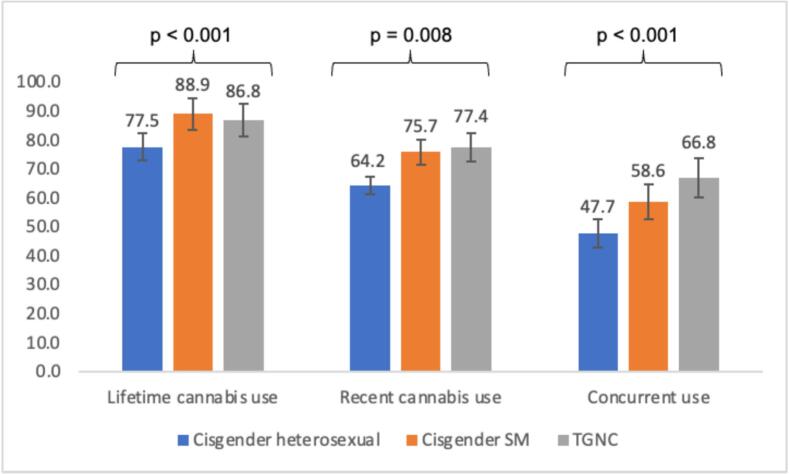
Prevalence of Cannabis Use Outcomes by Sexual Orientation and Gender Identity Among Participants Assigned Female at Birth (AFAB).

Bivariate differences in prevalence of the cannabis use outcomes among AFAB participants are presented in Fig. 1. Lifetime cannabis use differed significantly across groups, with 77.5 % of cisgender heterosexual, 88.9 % of cisgender SM, and 86.8 % of TGNC participants reporting lifetime cannabis use (p < 0.001). Recent cannabis use also differed significantly across groups, with 64.2 % of cisgender heterosexual, 75.7 % of cisgender SM, and 77.4 % of TGNC participants reporting recent use (p = 0.008). Finally, concurrent use of cannabis also differed significantly across groups, with 47.7 % of cisgender heterosexual, 58.6 % of cisgender SM, and 66.8 % of TGNC participants reporting co-use (p < 0.001).

**Fig. 2:**
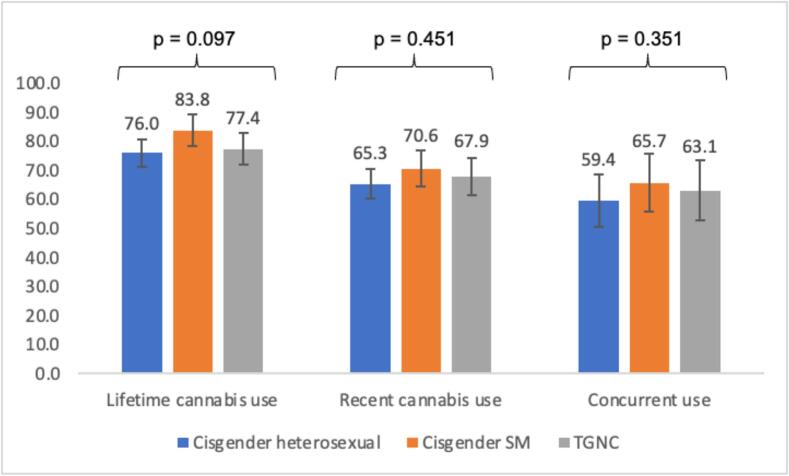
Prevalence of Cannabis Use Outcomes by Sexual Orientation and Gender Identity Among Participants Assigned Male at Birth (AMAB).

Bivariate differences in prevalence of the cannabis use outcomes among AMAB participants are presented in Fig. 2. Prevalence of lifetime cannabis use ranged from 76.0 % to 83.8 % across groups, though this difference only reached marginal statistical significance (p = 0.097). Prevalence of recent cannabis use ranged from 65.3 % to 70.6 % across groups, though these differences did not reach statistical significance (p = 0.451). Finally, prevalence rates of concurrent use ranged from 59.4 % to 63.1 % across groups, though these differences did not reach statistical significance (p = 0.351).

Differences in past 30-day blunt use across sexual/gender identity groups were also assessed across groups. Among AFAB participants, cisgender SM participants reported the highest prevalence of blunt use (44.6 %), followed by cisgender heterosexual (28.4 %) and TGNC participants (27.1 %; p = 0.004). Among AMAB participants, prevalence rates of blunt use ranged from 12.8 % (TGNC) to 56.8 % (cisgender heterosexual), though these differences did not reach statistical significance (p = 0.127).

Associations between sexual/gender identity and each cannabis use outcome, separately by assigned sex at birth are presented in Table 3. Model Set 1 presents models adjusted for sociodemographic covariates. Model Set 2 presents models adjusted for sociodemographic covariates, psychological distress, and social support. Consistent with bivariate estimates, in Model Set 1, both cisgender SM and TGNC AFAB participants had higher odds of lifetime cannabis use (cisgender SM: aOR = 2.38, 95 % CI = 1.54, 3.72; TGNC: aOR = 1.97, 95 % CI = 1.17, 3.40), recent cannabis use (cisgender SM: aOR = 1.72, 95 % CI = 1.29, 2.30; TGNC: aOR = 1.83, 95 % CI = 1.29, 2.61), and concurrent use of cannabis and tobacco products (cisgender SM: aOR = 1.49, 95 % CI = 1.11, 2.00; TGNC: aOR = 1.98, 95 % CI = 1.39, 2.82), compared to cisgender heterosexual AFAB participants. Among AMAB participants, however, no differences were noted between cisgender heterosexual, cisgender SM, and TGNC participants in odds of lifetime or recent use, or in concurrent use of cannabis and tobacco.Table 4..

**Table 3:** Associations between sexual and gender identity and cannabis use outcomes.

	**Lifetime Use**	**Recent Use**	**Concurrent Use**
	OR (95 % CI)	OR (95 % CI)	OR (95 % CI)
**Model Set 1**			
**Assigned Female at Birth**			
Cisgender heterosexual	Ref	Ref	Ref
Cisgender SM	2.38 (1.54, 3.72)***	1.72 (1.29, 2.30)***	1.49 (1.11, 2.00)*
TGNC	1.97 (1.17, 3.40)*	1.83 (1.29, 2.61)**	1.98 (1.39, 2.82)***
**Assigned Male at Birth**			
Cisgender heterosexual	Ref	Ref	Ref
Cisgender SM	1.52 (0.94, 2.48)	1.31 (0.93, 1.85)	1.40 (0.99, 1.97)
TGNC	0.85 (0.46, 1.63)	0.87 (0.55, 1.40)	0.95 (0.59, 1.52)
**Model Set 2**			
**Assigned Female at Birth**			
Cisgender heterosexual	Ref	Ref	Ref
Cisgender SM	2.14 (1.37, 3.36)**	1.59 (1.19, 2.14)**	1.40 (1.03, 1.89)
TGNC	1.69 (0.99, 2.96)	1.61 (1.12, 2.31)*	1.80 (1.25, 2.59)**
**Assigned Male at Birth**			
Cisgender heterosexual	Ref	Ref	Ref
Cisgender SM	1.37 (0.84, 2.27)	1.18 (0.83, 1.67)	1.32 (0.92, 1.87)
TGNC	0.74 (0.39, 1.43)	0.77 (0.48, 1.23)	0.87 (0.54, 1.40)

Note. Logistic regression models estimated lifetime cannabis use, while generalized ordinal logit models estimated the recent and concurrent use outcomes. For Model Set 1, all models controlled for race/ethnicity, country of birth, educational attainment, employment, housing stability, marital status, and age. For Model Set 2, all models controlled for psychological distress and social support, in addition to Model Set 1 covariates. ***p < 0.001, **p < 0.01, *p < 0.05. Benjamini-Hochberg-adjusted p-values were used.

**Table 4:** Tobacco products used among those reporting concurrent cannabis use and tobacco use.

	**Cigarette Use (%)**	**E-Cigarette Use (%)**	**Other Tobacco Product Use (%)**	**Multiple Tobacco Product Use (%)**
**Assigned Female at Birth**				
Cisgender heterosexual	52.1	72.2	22.2	41.0
Cisgender SM	63.1	68.5	24.3	44.1
TGNC	74.0	66.1	29.1	55.1
P-Value	0.001	0.545	0.405	0.049
**Assigned Male at Birth**				
Cisgender heterosexual	61.7	72.1	35.5	55.2
Cisgender SM	63.4	76.1	23.1	50.0
TGNC	77.4	62.3	32.1	50.9
P-Value	0.105	0.163	0.059	0.634

Note. Differences in tobacco product use were tested across sexual/gender identities using chi-square tests. Tobacco use categories are not mutually exclusive.

Results were fairly consistent in Model Set 2. Cisgender SM AFAB had higher odds of lifetime (aOR = 2.14, 95 % CI = 1.37, 3.36) and recent (aOR = 1.59, 95 % CI = 1.19, 2.14) cannabis use, and TGNC AFAB had higher odds of recent cannabis (aOR = 1.61, 95 % CI = 1.12, 2.31) and concurrent cannabis and tobacco use (aOR = 1.80, 95 % CI = 1.25, 2.59), compared to cisgender heterosexual AFAB. No differences were noted between cisgender heterosexual, cisgender SM, and TGNC AMAB.

### 3.3 Tobacco products used among those reporting concurrent cannabis and tobacco use

The specific tobacco products used, among those reporting concurrent use of both cannabis and tobacco products are reported in Table 3. Among AFAB participants reporting concurrent use, identity-based differences in use of cigarettes were observed, with TGNC participants reporting the highest prevalence of use (74.0 %), followed by cisgender SM (63.1 %) and cisgender heterosexual participants (52.1 %; p = 0.001). Differences in use of multiple tobacco product types were also observed, with TGNC participants reporting the highest prevalence of use (55.1 %), followed by cisgender SM (44.1 %) and cisgender heterosexual participants (41.0 %; p = 0.049). No other differences in the specific tobacco products used were observed between groups of AFAB reporting concurrent use. Similarly, among AMAB reporting concurrent cannabis and tobacco use, no differences were noted between groups in the specific tobacco products used.

## 4 Discussion

We found high rates of cannabis and tobacco co-use across our sample of both SGM and non-SGM emerging adults. This aligns with the growing body of research on the increasing prevalence of co-use among young people, generally (Cohn et al., 2019, Wheldon et al., 2023). For example, 21.3 % of young adults (ages 18–24) in the PATH study report past-month co-use of cannabis and at least one tobacco product (Cohn et al., 2019). However, our prevalence estimates were considerably higher; given our study’s inclusion criterion of past 30-day tobacco use, our prevalence estimates should not be interpreted as the “true” prevalence of cannabis use or co-use among emerging adults, but as prevalence estimates of cannabis use and co-use among emerging adults *who use tobacco*. Broadly, our findings suggests that emerging adults who use tobacco are also at high risk for co-using cannabis.

Our study also adds to research showing that SGM emerging adults use cannabis at higher rates than do cisgender heterosexual emerging adults (Dai, 2017, Delahanty et al., 2019, Dunbar et al., 2022, Harlow et al., 2023, Krueger et al., 2020, Romm et al., 2024, Romm et al., 2023, Sawyer et al., 2022). While there is considerably less research on cannabis and tobacco co-use among SGM, or on SGM disparities in co-use, there appear to be considerable differences in co-use on the basis of sexual identity. In a study of adults from the 2022 National Survey on Drug Use and Health, gay/lesbian (14.8 %) and bisexual (22.8 %) AFAB respondents reported past-month tobacco and cannabis co-use, compared to only 4.6 % of heterosexual AFAB respondents. These differences were narrower among AMAB, where 10.1 % of heterosexual, 14.6 % of gay, and 18.8 % of bisexual respondents reported co-use (Lee et al, 2025). We also found stark SGM disparities in cannabis and tobacco co-use in our sample, and particularly among AFAB. Specifically, cisgender SM and TGNC AFAB participants in our sample reported higher rates of lifetime, recent, and concurrent use than did cisgender heterosexual AFAB. There were also identity-based differences in the specific tobacco products used among AFAB participants reporting concurrent cannabis and tobacco use, with TGNC participants reporting the greatest prevalence concurrent cannabis and cigarette use, as well as concurrent cannabis and multiple tobacco product use, compared to cisgender heterosexual AFAB, who had the lowest prevalence of each outcome.

Notably, we conversely found no significant differences in co-use across AMAB participant groups. These findings partially conflict with the Lee et al. (2025) study, which did find higher rates of cannabis and tobacco co-use among sexual minority AMAB adults, compared to heterosexual AMAB adults, though those differences were less pronounced than among AFAB participants. While we are unable to test reasons for the “lack” of disparities noted among AMAB in our sample, there are several plausible hypotheses. First, it is possible that the absence of significant differences among AMAB in our sample is due to reduced analytic power (e.g., our sample contained 84 TGNC AMAB). However, the consistency of this pattern across cannabis use outcomes strengthens our confidence in our findings. Second, our sampling design may also have contributed: all participants used tobacco (a study inclusion criterion) and resided in California, a state with robust tobacco regulatory policies. It is possible that in this context, tobacco use is a particularly strong predictor of cannabis use. Third, the disparities observed among AFAB participants may also be partly explained by the relatively low rates of co– and concurrent use among cisgender heterosexual AFAB, in contrast to higher rates among cisgender SM and TGNC AFAB, as well as across all AMAB groups. Therefore, the lack of a noted disparity among AMAB groups may reflect the relatively high rates of co– and concurrent use among heterosexual AMAB, rather than relatively low rates of use among sexual or gender minorities within that group.

Regardless, there remains a lack of SGM-specific cannabis use prevention and harm reduction interventions. Prior research has called for the need for tailored interventions that are responsive to SGM-specific needs (e.g., substance use interventions which promote acceptance and connection with other SGM people) (Dyar, 2022, Flaherty, 2025). Our findings underscore this need and suggest that interventions may need to be tailored even more specifically to AFAB SGM emerging adults. Additionally, given the elevated rates of co-use among all AMAB participants, there may also be value in developing interventions responsive to the needs of AMAB emerging adults, irrespective of SGM status.

While we did account for psychological distress and social support in multivariable analyses, the focus of this study was not on the social/behavioral mechanisms driving disparities in use (Jauregui et al., 2024). To identify appropriate interventions, future studies should explore the social/behavioral factors underlying differences in cannabis and tobacco co-use between AFAB and AMAB participants. Minority stress, or chronic social stress derived from one’s minoritized identity (e.g., stigma, discrimination, invalidation), is a commonly proposed mechanism contributing to SGM young persons’ use of substances to cope, and thus higher rates of use for SGM young people, compared to their non-SGM peers (Goldbach et al., 2015, Goldbach et al., 2014, Threeton et al., 2024). This is a plausible explanation for some of our findings (and which reinforces the need for SGM-tailored interventions). However, minority stress may not fully explain why disparities were found among AFAB participants, but not AMAB participants. Pro-cannabis marketing may represent another mechanism underlying sex differences in SGM cannabis use disparities. Prior research has shown that young women (vs. young men) and gender minority emerging adults (vs. cisgender emerging adults) report seeing cannabis advertisements at higher rates (Krueger et al., 2021a). Given the rapid increase in pro-cannabis marketing following recreational cannabis legalization in California (Marijuana Policy Project, 2021), where our study was situated, reducing SGM-targeted marketing represents a potential avenue for regulatory efforts to reduce AFAB disparities in co-use of cannabis and tobacco. Additional research is needed to explore this and other possible approaches for reducing SGM AFAB disparities in cannabis and tobacco co-use.

### 4.1 Limitations

Our findings should be interpreted in light of several limitations. First, this survey was conducted with a convenience sample of emerging adults from California. While specific efforts were made to recruit a demographically diverse sample, these findings may not be representative of emerging adults across the United States. Second, to participate in the survey, participants had to report recent (past 30-day) tobacco use. Thus, we were unable to assess SGM differences in cannabis use in isolation of tobacco use, and this study focused specifically on co-use and concurrent of cannabis and tobacco. Third, to increase analytic power, all SM identities were collapsed into a single analytic group. Gender minority participants were also collapsed into a single group, regardless of the sexual identity they endorsed. This approach may have masked differences in cannabis use across specific sexual (e.g., lesbian vs. bisexual vs. pansexual) and gender (e.g., transgender vs. non-binary) identities, as has been seen in other studies (e.g., Lee et al., 2025). However, this approach also allowed us to disaggregate sexual and gender identity differences in cannabis and tobacco co-use by sex assigned at birth (e.g., between AFAB and AMAB). Finally, it is important to note that the data used in this study were collected in 2020–2021 during the COVID-19 pandemic and associated lockdowns. Numerous studies have shown that substance use behaviors among young people changed in complex ways during this period, with initial overall reductions in alcohol and tobacco use during the early pandemic but increases among those already engaging in substance use, potentially exacerbating existing disparities among marginalized young people (Hussong et al., 2023, Sylvestre et al., 2022, Krueger et al., 2021b). While our results highlight expected disparities in the use and co-use of cannabis and tobacco that likely existed before and extend beyond the pandemic, the prevalence point estimates should be interpreted with caution.

## 5 Conclusions

This study compared rates of co-use of cannabis and tobacco products across dimensions of sexual identity, gender identity, and sex assigned at birth. Broadly, we found wide-ranging disparities in co-use between AFAB cisgender heterosexual, cisgender SM, and TGNC participants, but relatively few differences among AMAB participants. These findings highlight potential areas for further study, and for tailoring interventions for reducing SGM cannabis and tobacco co-use disparities.

## CRediT authorship contribution statement

**Evan A. Krueger:** Supervision, Writing – original draft, Conceptualization, Writing – review & editing. **Luisita Cordero:** Writing – review & editing, Data curation, Formal analysis. **Chenglin Hong:** Writing – review & editing. **Risa Flynn:** Writing – review & editing. **Ian W. Holloway:** Writing – review & editing, Conceptualization, Funding acquisition.

## Declaration of competing interest

The authors declare that they have no known competing financial interests or personal relationships that could have appeared to influence the work reported in this paper.

## Data Availability

Data will be made available on request.
